# Association between baseline pulse pressure and hospital mortality in non-traumatic subarachnoid hemorrhage patients: a retrospective cohort study

**DOI:** 10.3389/fneur.2023.1176546

**Published:** 2023-07-17

**Authors:** Jiuling Liu, Shu Wang, Lin Ji, Xiaoqing Wang, Hang Zhao

**Affiliations:** ^1^Department of Neurology, Nanjing BenQ Medical Center, The Affiliated BenQ Hospital of Nanjing Medical University, Nanjing, China; ^2^Department of Anesthesiology, The Yancheng School of Clinical Medicine of Nanjing Medical University (Yancheng Third People’s Hospital), Yancheng, Jiangsu Province, China; ^3^Department of Anaesthesiology, Zhongshan Hospital, Fudan University, Shanghai, China

**Keywords:** pulse pressure, non-traumatic subarachnoid hemorrhage, hospital mortality, retrospective, MIMIC

## Abstract

**Background and purpose:**

Previous studies have described an association between pulse pressure (PP) level and mortality in stroke patients. Evidence of associations between PP level and the risk of mortality remains unknown in non-traumatic subarachnoid hemorrhage (SAH) patients. We aimed to explore the relationship between the baseline PP level and hospital mortality.

**Methods:**

This cohort study of 693 non-traumatic SAH adults used Medical Information Mart for Intensive Care (MIMIC-IV) data from 2008–2019 admissions to Intensive Care Unit (ICU). PP level was calculated as the first value after admission to the ICU. The endpoint of the study was in-hospital mortality. Cox proportional hazards models were utilized to analyze the association between baseline PP level and hospital mortality. Restricted Cubic Splines (RCS) analysis was utilized to determine the relationship curve between hospital mortality and PP level and examine the threshold saturation effect. We further applied Kaplan–Meier survival curve analysis to examine the consistency of these correlations. The interaction test was used to identify subgroups with differences.

**Results:**

The mean age of the study population was 58.8 ± 14.6 years, and 304 (43.9%) of participants were female. When baseline PP level was assessed in quartiles, compared to the reference group (Q1 ≤ 56 mmHg), the adjusted hazard ratio (HR) in Q2 (57–68 mmHg), Q3(69–82 mmHg), Q4 (≥83 mmHg) were 0.55 (95% CI: 0.33–0.93, *p* = 0.026), 0.99 (95% CI, 0.62–1.59, *p* = 0.966), and 0.99 (95% CI: 0.62–1.59, *p* = 0.954), respectively. In the threshold analysis, for every 5 mmHg increase in PP level, there was an 18.2% decrease in hospital mortality (adjusted HR, 0.818; 95% CI, 0.738–0.907; *p* = 0.0001) in those with PP level less than 60 mmHg, and a 7.7% increase in hospital mortality (adjusted HR, 1.077; 95% CI, 1.018–1.139; *p* = 0.0096) in those with PP level was 60 mmHg or higher.

**Conclusion:**

For patients with non-traumatic SAH, the association between baseline PP and risk of hospital mortality was non-linear, with an inflection point at 60 mmHg and a minimal risk at 57 to 68 mmHg (Q2) of baseline PP level.

## Introduction

Despite advances in critical care, non-traumatic subarachnoid hemorrhage (SAH) remains a devastating form of stroke with a high mortality rate (1). It is estimated that up to 40% of non-traumatic SAH patients die in the hospital (2). Given the mortality risk of subarachnoid hemorrhage, it highlights the critical need for non-invasive and inexpensive tests to help identify patients at increased risk for mortality and further optimize the treatment.

Among the various factors identified that may increase the mortality risk of non-traumatic SAH, blood pressure (BP) is one of the most important, as it is essential for maintaining adequate cerebral perfusion pressure (3, 4). In addition to the systolic and diastolic stable components, BP is well characterized by its pulsatile nature and is estimated by pulse pressure (PP), which can either become broader or narrower. The main determinants of PP are per-pulse output and aortic resistance. It is inversely related to elastic properties of arterial wall, i.e., higher the PP level, stiffer the large arterial wall, which is reduces aortic compliance (5). PP also reflects fluid responsiveness, stroke, and left ventricular function and volume. Notably, a recent systematic review and meta-analysis of 11 studies revealed that a high PP level was associated with an increased risk of stroke (6); Furthermore, there is mounting evidence that PP level was strongly related to mortality in patients with acute intracerebral hemorrhage and ischemic stroke, as well as increasing stroke recurrence (5, 7–12). A previous study including 156 patients with SAH demonstrated no statistically significant association between admission PP and hospital mortality (13). However, evidence of associations between PP level and the risk of mortality remains unknown in non-traumatic subarachnoid hemorrhage (SAH) patients.

The Medical Information Mart for Intensive Care (MIMIC-IV) database of ICU allowed us to assess the associations of baseline PP level with non-traumatic SAH. We aimed to explore the association between baseline PP level and hospital mortality.

## Materials and methods

### Data source

The MIMIC-IV maintains a standardized and publicly accessible ICU database, an extensive single-center database, which includes 50,048 ICU admissions at Boston’s Beth Israel Deaconess Medical Center (BIDMC) between 2008 and 2019. To extract data, all authors completed the National Institutes of Health’s web-based course “Protecting Human Research Participants” (certification number: 46264188) and obtained authorization to access the database. The study was carried out following the Helsinki Declaration guidelines and was reviewed and approved by the Massachusetts Institute of Technology and the Institutional Review Board of Beth Israel Deaconess Medical Center (BIDMC, Boston, MA, United States). All data were de-identified to protect patient privacy, and the need for informed consent was waived. This study follows the Strengthening the Reporting of Observational Studies in Epidemiology (STROBE) statement (14).

### Study population

We conducted a retrospective analysis of 693 non-traumatic SAH database from an online international database from 2008 to 2019, the Medical Information Mart for Intensive Care (MIMIC-IV) (15). Data includes demographics, clinical parameters, clinical laboratory tests, intervention, medical history, and medical data. Among them, 693 patients with non-traumatic SAH were selected based on the record of ICD-9 code 430, and ICD-10 codes I60, I600 to I6012, I6000 to I6002, I6020 to I6022, I6030 to I6032, I6050 to I6052. Patients who met the following criteria were included: (1) diagnosed as non-traumatic SAH at ICU admission; (2) aged ≥18 years; (3) had their first ICU admission. The exclusion criteria were as follows: (1) missing admission systolic blood pressure (SBP) or diastolic blood pressure (DBP) data from invasive measurements; (2) dying within 24 h of ICU admission. Finally, 693 patients (389 male and 304 female) were enrolled, and complete baseline data was collected.

### Data extraction

All relevant variables were taken from the medical record using Structured Query Language (SQL) with PostgreSQL in the MIMIC-IV database. As previously stated, we accumulated the following patient characteristics: age (≥18 years), sex (389 male and 304 female), ethnicity (white, or others), SBP, DBP, heart rate, respiratory rate (RR), percutaneous oxygen saturation (SpO2), Glasgow Coma Scale (GCS) score, Charlson comorbidity index, comorbidities (including myocardial infarction, congestive heart failure, chronic pulmonary disease, diabetes, hypertension, paraplegia, and sepsis), white blood cell (WBC), platelet, hemoglobin, glucose, sodium, potassium, BUN, creatinine and therapies performed during the first day of ICU and hospital stay (including the use of norepinephrine, vasopressin, dopamine, nicardipine, nimodipine, embolization of aneurysm, and clipping of aneurysm). In addition, the ICD-9 discharge diagnostic codes were examined for the hypertension diagnosis.

### Baseline PP level measurement and definition of outcomes

Baseline arterial blood pressure defined as the first measurement recorded on admission to the ICU. These values were invasively obtained through an arterial catheter. The pulse pressure (PP; first SBP minus first DBP) were calculated for each patient (16, 17). The primary endpoint was all-cause hospital mortality, with secondary endpoints including 28 day post-admission and all-cause ICU mortality.

### Statistical analyses

The means ± SD or median and interquartile ranges (IQR) were used to describe continuous variables. Numbers and percentages were used to represent categorical data. The difference between the admission PP level quartile was compared using one-way ANOVA for continuous data and chi-squared tests for categorical variables (18).

Next, we used cox proportional hazards models to investigate the relationship between PP level at baseline and hospital mortality. We performed three models. We adjusted for no covariates in the crude model. We adjusted for age, sex, and ethnicity in Model I. In the Model II, we adjusted for age, sex, ethnicity, RR, WBC, platelets, GCS score, Charlson Comorbidity Index, congestive heart failure, myocardial infarction, hypertension, sepsis, vasoactive drugs, nicardipine, nimodipine, and aneurysm embolization. The results are presented as hazard ratios (HRs) with 95% confidence intervals (CIs). We chose these confounders based on their relationship with the clinical outcomes of interest or significant changes in effect estimates of more than 10% (19).

Threshold analysis in the association of baseline PP level with hospital mortality was conducted with a two-piece-wise Cox regression model using restricted cubic spline analysis (RCS) (20, 21). The inflection point for the baseline PP level was identified using “exploratory” analyses, which involved moving the trial turning point along a pre-defined interval and selecting the one with the maximum model likelihood. A log-likelihood ratio test was also performed, and the one-line linear regression model was compared to the two-piece-wise linear model (20). We used the bootstrap resampling method to calculate the 95% CI for the turning point, as described in the previous analysis (21–23). The Kaplan–Meier method was used to estimate survival, and any differences in survival were assessed using stratified log-rank tests and bootstrap resampling method (20).

Furthermore, we also performed sensitivity analyses to confirm the stability of our study. Interactions and stratified analyses were conducted using sex (male vs. female), myocardial infarction (no vs. yes), congestive heart failure (no vs. yes), hypertension (no vs. yes), diabetes (no vs. yes), endovascular therapy of aneurysm (no vs. yes), and GCS (<8 and ≥8) results. Cox proportional hazards models were used to assess heterogeneity across subgroups, and likelihood ratio testing was used to examine interactions between subgroups and baseline PP level (18). We used the predicted mean matching method to replace missing values in the data for the missing dataset (24). Supplementary Table S1 displays the details of the missing data.

In all analyses, *p* < 0.05 (two-sided) was considered statistically significant. All data analyses were carried out using R 3.6.1^1^ and the EmpowerStats package (www.empowerstats.com, X&Y solutions, Inc. Boston MA) (25, 26).

## Results

### Baseline characteristics

**Figure 1:**
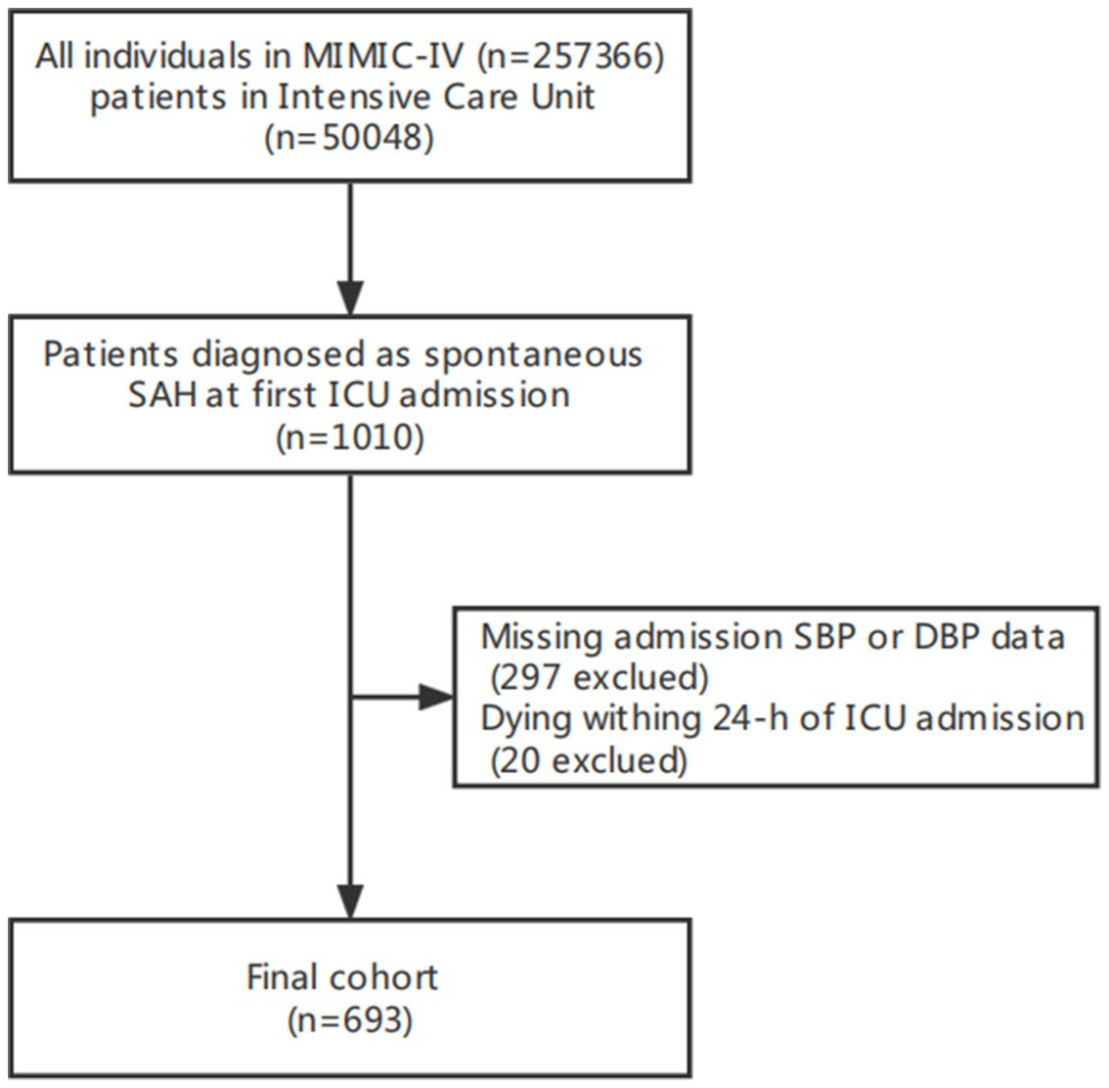
Flow chart of study population. MIMC-IV, medical information mart for intensive care IV; ICU, intensive care unit.

Our study included 693 non-traumatic SAH participants with complete SBP and DBP recorded through invasive measurements from the MIMIC-IV database (Figure 1). The mean age of the study participants was 58.8 ± 14.6 years, and 304 (43.9%) of participants were female. Table 1 describes the baseline characteristics of included patients through the quartiles of the baseline PP level (Q1 < 56 mmHg, 57 ≤ Q2 < 68 mmHg, 69 ≤ Q3 < 82 mmHg, Q4 ≥ 83 mmHg) in this study.

**Table 1:** Population characteristics by quartiles of baseline pulse pressure.

Variables	Pulse pressure (mmHg)					*p* value
	Total	Q1 (≤56)	Q2 (57–68)	Q3 (69–82)	Q4 (≥83)	
No of individuals	693	168	165	185	175	
Age, years	58.8 ± 14.6	54.7 ± 14.1	56.4 ± 13.5	60.0 ± 14.9	64.0 ± 14.0	<0.001
Female, *n*(%)	304 (43.9)	80 (47.6)	68 (41.2)	79 (42.7)	77 (44)	0.674
White race, *n*(%)	293 (42.3)	75 (44.6)	59 (35.8)	77 (41.6)	82 (46.9)	0.187
Vital signs						
SBP, mmHg	136.9 ± 24.7	110.4 ± 17.0	130.1 ± 13.3	142.1 ± 13.6	163.1 ± 18.6	<0.001
DBP, mmHg	67.0 ± 13.6	65.7 ± 14.6	67.4 ± 12.7	67.1 ± 13.2	67.9 ± 13.9	0.510
MBP, mmHg	82.6 ± 8.3	82.4 ± 8.6	82.9 ± 8.6	83.7 ± 8.3	81.6 ± 7.7	0.113
Heart rate, beats/min	79.0 ± 12.9	84.7 ± 13.9	78.0 ± 12.1	76.8 ± 12.9	76.7 ± 11.0	<0.001
RR, times/min	18.3 ± 3.2	19.2 ± 3.5	18.4 ± 3.6	17.5 ± 3.0	18.1 ± 2.6	<0.001
Temperature, °C	37.0 ± 0.6	37.0 ± 0.7	37.1 ± 0.4	37.0 ± 0.4	37.1 ± 0.6	0.353
SpO2, %	97.5 ± 2.0	97.8 ± 2.3	97.6 ± 1.8	97.4 ± 1.9	97.4 ± 2.0	0.270
Comorbidities, *n* (%)						
Myocardial infarction	58 (8.4)	23 (13.7)	5 (3)	13 (7)	17 (9.7)	0.004
Congestive heart failure	50 (7.2)	15 (8.9)	5 (3)	12 (6.5)	18 (10.3)	0.053
Chronic pulmonary disease	108 (15.6)	26 (15.5)	23 (13.9)	34 (18.4)	25 (14.3)	0.645
Diabetes	88 (12.7)	16 (9.5)	20 (12.1)	19 (10.3)	33 (18.9)	0.036
Hypertension	346 (49.9)	69 (41.1)	77 (46.7)	95 (51.4)	105 (60)	0.004
Paraplegia	100 (14.4)	21 (12.5)	25 (15.2)	27 (14.6)	27 (15.4)	0.868
Sepsis	393 (56.7)	115 (68.5)	82 (49.7)	91 (49.2)	105 (60)	<0.001
Charlson comorbidity index	4.0 (3.0, 6.0)	4.0 (3.0, 6.0)	4.0 (3.0, 6.0)	4.0 (3.0, 6.0)	5.0 (4.0, 7.0)	<0.001
Laboratory results						
WBC,10^9^/L	14.5 ± 6.6	16.2 ± 7.7	14.4 ± 6.1	13.6 ± 5.1	14.1 ± 7.1	0.001
Platelets,10^9^/L	239.4 ± 90.9	242.0 ± 97.9	243.4 ± 83.4	237.7 ± 92.0	235.1 ± 89.9	0.825
Hemoglobin, g/dL	13.0 ± 1.9	12.9 ± 2.0	13.2 ± 2.0	13.1 ± 1.9	12.7 ± 1.8	0.093
Glucose, mg/dL	142.2 ± 37.9	145.9 ± 46.5	140.0 ± 34.5	138.0 ± 34.1	145.3 ± 35.3	0.133
Sodium, mg/dL	141.8 ± 5.4	142.2 ± 6.3	141.9 ± 4.6	141.4 ± 5.3	141.6 ± 5.2	0.580
Potassium, mg/dL	4.3 ± 0.8	4.4 ± 0.9	4.2 ± 0.7	4.2 ± 0.8	4.2 ± 0.8	0.252
BUN, mg/dL	17.9 ± 10.6	18.4 ± 12.2	16.0 ± 8.4	18.2 ± 11.2	18.8 ± 9.8	0.077
Creatinine, mg/dL	1.0 ± 1.0	1.0 ± 0.8	0.9 ± 0.5	1.1 ± 0.8	1.1 ± 1.4	0.347
Therapy, *n* (%)						
Norepinephrine	180 (26.0)	66 (39.3)	48 (29.1)	31 (16.8)	35 (20)	<0.001
Vasopressin	78 (11.3)	35 (20.8)	20 (12.1)	15 (8.1)	8 (4.6)	<0.001
Dopamine	13 (1.9)	8 (4.8)	2 (1.2)	1 (0.5)	2 (1.1)	0.036
Nicadipine	495 (71.4)	105 (62.5)	115 (69.7)	136 (73.5)	139 (79.4)	0.005
Nimodipine	484 (69.8)	105 (62.5)	120 (72.7)	128 (69.2)	131 (74.9)	0.069
Embolization of aneurysm	202 (29.1)	39 (23.2)	64 (38.8)	54 (29.2)	45 (25.7)	0.010
Clipping of aneurysm	36 (5.2)	10 (6)	9 (5.5)	11 (5.9)	6 (3.4)	0.673
Scoring systems						
GCS	11.0 (7.0, 14.0)	9.0 (4.8, 14.0)	13.0 (6.0, 14.0)	13.0 (8.0, 14.0)	11.0 (7.0, 14.0)	0.006
Outcomes						
28 day mortality	132 (19.0)	43 (25.6)	23 (13.9)	30 (16.2)	36 (20.6)	0.034
ICU mortality	110 (15.9)	38 (22.6)	18 (10.9)	21 (11.4)	33 (18.9)	0.005
Hospital mortality	142 (20.5)	47 (28)	24 (14.5)	33 (17.8)	38 (21.7)	0.016

SBP, systolic blood pressure; DBP, diastolic blood pressure; MBP, mean blood pressure; RR, respiratory rate; SpO2, percutaneous oxygen saturation; WBC, white blood cell; GCS, Glasgow coma score; ICU, intensive care unit; SAH, subarachnoid hemorrhage.

Participants with higher levels of PP tended to be older; more likely to have higher systolic blood pressure; comorbid with myocardial infarction, diabetes, sepsis; however participants with lower levels of PP more likely to have lower GCS; to be more likely use norepinephrine, vasopressin, and dopamine drugs. (All *p* < 0.05).

### Association between baseline PP level and hospital mortality

The univariate analysis demonstrated that age, ethnicity, RR, myocardial infarction, congestive heart failure, Charlson comorbidity index, WBC, platelets, glucose, sodium, potassium, BUN, creatinine, norepinephrine, vasopressin, nicadipine, nimodipine, GCS and embolization of aneurysm were associated with hospital mortality (all *p* < 0.05) (Supplementary Table S2).

**Figure 2:**
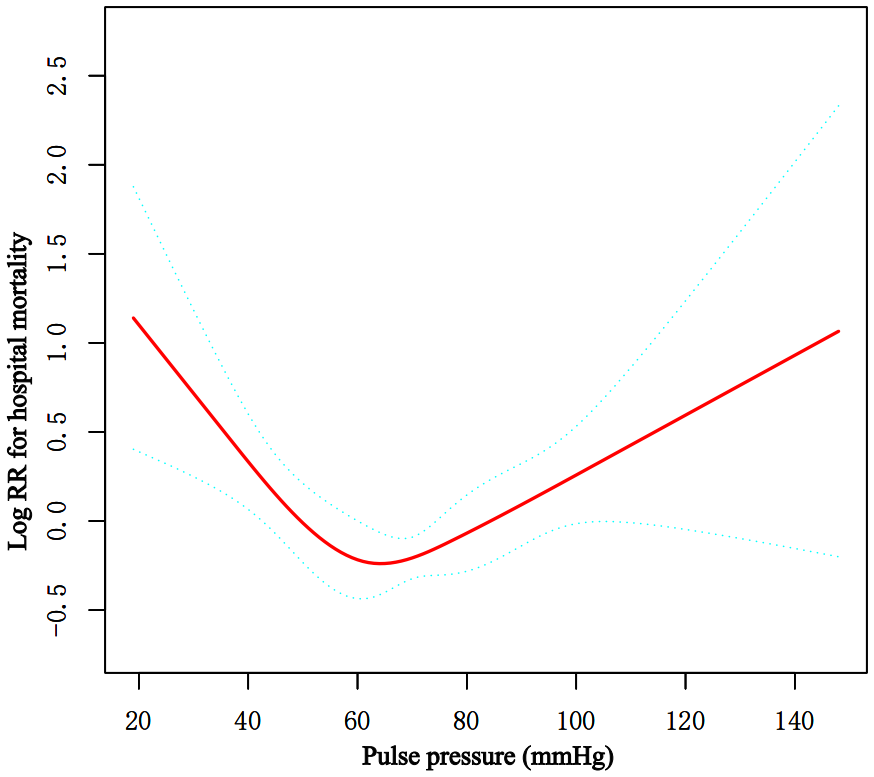
Associations between the baseline PP level and in-hospital mortality in critically ill patients with non-traumatic SAH. A threshold, nonlinear association between the baseline PP level and hospital mortality was found in restricted cubic spline (RCS). Solid rad line represents the smooth curve fit between variables. Green bands represent the 95% of confidence interval from the fit. Adjusted for age, sex, ethnicity, RR, WBC, platelet, GCS score, Charlson Comorbidity Index, congestive heart failure, myocardial infarction, hypertension, sepsis, vasoactive drugs, nicardipine, nimodipine, embolization of aneurysms. PP, pulse pressure; SAH, subarachnoid hemorrhage.

Multiple Cox proportional hazard regression analyses were used to investigate the relationship between baseline PP level and hospital mortality in Table 2. We performed three model. We adjusted for no covariates in the crude model. We adjusted for age, sex, and ethnicity in Model I. We adjusted for age, gender, ethnicity, RR, WBC, platelets, GCS score, Charlson Comorbidity Index, congestive heart failure, myocardial infarction, hypertension, sepsis, vasoactive drugs, nicardipine, nimodipine, and aneurysm embolization in the Model II. In any of the three models (all *p* > 0.05), there was no significant relationship between the baseline PP level and hospital mortality when it was used as a continuous variable. Overall, the association between baseline PP Level and hospital mortality followed a nonlinear curve using a smooth curve function in Figure 2 (*p* = 0.041). Accordingly, when baseline PP Level was assessed in quartiles and compared with Q1 (<56 mmHg), the risk of hospital mortality was lower for Q2 (57 to ≤68 mmHg: adjusted HR, 0.99; 95% CI, 0.62–0.93; *p* = 0.026), Q3 (69 to ≤82 mmHg: adjusted HR, 0.99; 95% CI, 0.62–1.59; *p* = 0.966), and Q4 (≥83 mmHg: adjusted HR, 0.99; 95% CI, 0.62–1.57; *p* = 0.954) (Table 2). The lowest risk of hospital mortality was found in those in Q2. When quartiles were combined in a further exploratory study, a significantly higher risk of hospital mortality was found among patients in Q1 (<56 mmHg: adjusted HR, 1.85; 95% CI, 1.11–3.10; *p* = 0.019) and in Q3–4 (≥69 mmHg: adjusted HR, 1.78; 95% CI, 1.08–2.93; *p* = 0.024) compared to those in Q2 (57 to ≤68 mmHg) (Table 2).

**Table 2:** Relationship between baseline pulse pressure and hospital mortality stratified by quartiles and combined quartiles.

Pulse pressure, mmHg	Total	Event (*n*, %)	Crude		Model I		Model II	
			HR (95%CI)	Value of *p*	HR (95%CI)	Value of *p*	HR (95%CI)	Value of *p*
Continuous	693	142 (20.5)	1.00 (0.99, 1.01)	0.503	1.00 (0.99, 1.01)	0.563	0.99 (0.99, 1.00)	0.231
Quartiles								
Q1 (≤56 mmHg)	168	47 (28.0)	1 (Ref)		1 (Ref)		1 (Ref)	
Q2 (57–68 mmHg)	165	24 (14.5)	0.51 (0.31, 0.84)	0.009	0.50 (0.30, 0.82)	0.006	0.55 (0.33, 0.93)	0.026
Q3 (69–82 mmHg)	185	33 (17.8)	0.72 (0.46, 1.13)	0.158	0.62 (0.40, 0.98)	0.041	0.99 (0.62, 1.59)	0.966
Q4 (≥83 mmHg)	175	38 (21.7)	0.89 (0.58, 1.37)	0.604	0.67 (0.43,1.05)	0.078	0.99 (0.62, 1.57)	0.954
Categories								
Q1 (≤56 mmHg)	168	47 (28.0)	1.95 (1.19, 3.22)	0.009	2.02 (1.22, 3.34)	0.006	1.85 (1.11, 3.10)	0.019
Q2 (57–68 mmHg)	165	24 (14.5)	1(Ref)		1(Ref)		1(Ref)	
Q3–4 (≥69 mmHg)	360	71(39.5)	1.57 (0.98, 2.52)	0.059	1.31 (0.81, 2.11)	0.271	1.78 (1.08, 2.93)	0.024

HR, hazard ratio; Q, quartile. Crude: no covariates were adjusted; Model I: adjusted for age, sex, and ethnicity. Model II: adjusted for age, sex, ethnicity, RR, WBC, platelet, GCS score, Charlson Comorbidity Index, congestive heart failure, myocardial infarction, hypertension, sepsis, vasoactive drugs, nicardipine, nimodipine, embolization of aneurysms. CI, confidence interval; HR, hazards ratio; Ref, reference.

**Figure 3:**
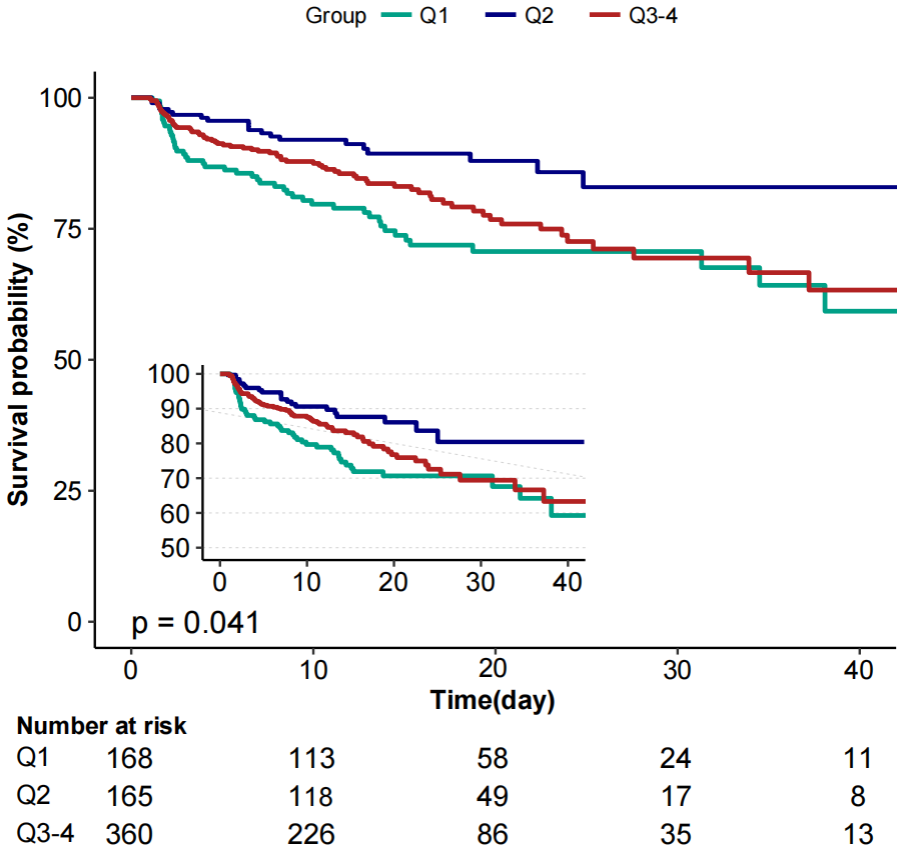
Kaplan–Meier survival curves for critically ill patients with non-traumatic SAH based on baseline PP level. *X*-axis: survival time (days). *Y*-axis: survival probability. PP, pulse pressure; SAH, subarachnoid hemorrhage.

Moreover, the K-M curves contrasting the three groups were displayed in Figure 3. The figure indicated that the survival rate of group Q2 was higher than groups Q1and Q3 (*p* = 0.041).

### The results of the two-piecewise linear regression model

Piecewise multivariate Cox regression with two different slopes was used to confirm the relationship between PP at baseline and in-hospital mortality. In the threshold analysis, for every 5 mmHg increase in PP level, there was an 18.2% decrease in hospital mortality (adjusted HR, 0.818; 95% CI, 0.738–0.907; *p* = 0.0001) in those with PP level 60 mmHg, and a 7.7% increase in hospital mortality (adjusted HR, 1.077; 95% CI, 1.018–1.139; *p* = 0.0096) in those with PP level 60 mmHg or higher. The linear regression model and a two-piecewise linear regression model were compared, and the *p* value of the log-likelihood ratio test was <0.001 (Table 3 and Figure 2).

**Table 3:** Threshold effect analysis of pulse pressure and hospital mortality.

Models	Per-1 mmHg increase		Per-5 mmHg increase	
	HR (95%CI)	*p* value	HR (95%CI)	*p* value
Model I				
One line effect	0.999 (0.990, 1.007)	0.7778	0.994 (0.952, 1.037)	0.7778
Model II				
Turning point (K, mmHg)	60		12	
pulse pressure < *k*	0.964 (0.946, 0.983)	0.0001	0.818 (0.738, 0.907)	0.0001
pulse pressure ≥ *k*	1.016 (1.004, 1.028)	0.0096	1.077 (1.018, 1.139)	0.0096
*P* value for LRT test*		<0.001		<0.001

Data were presented as HR (95% CI) *p* values; Model I, linear analysis; and Model II, non-linear analysis. Adjusted for age, sex, ethnicity, RR, WBC, platelet, GCS score, Charlson Comorbidity Index, congestive heart failure, myocardial infarction, hypertension, sepsis, vasoactive drugs, nicardipine, nimodipine, embolization of aneurysms. CI, confidence interval; HR, hazards ratio; LRT, logarithm likelihood ratio test. *P < 0.05 indicates that Model II is significantly different from Model I.

### Subgroup analysis

We performed further stratified and interaction analysis to assess the association between baseline PP level (Q1 vs. Q2 vs. Q3–4) and the risk of hospital mortality in various subgroups (Table 4): sex (male vs. female), myocardial infarction (no vs. yes), congestive heart failure (no vs. yes), hypertension (no vs. yes), diabetes (no vs. yes), endovascular therapy of aneurysm (no vs. yes), GCS (<8 and ≥8) results. The non-linear association was consistent across all subgroups, except for diabetes. The stratified analysis demonstrated association between baseline PP level and the risk of hospital mortality subjects without diabetes [(adjusted HR, 1.912; 95% CI, 1.090–3.353) vs. (1) vs. (adjusted HR, 1.601; 95% CI, 0.920–2.785)], with diabetes [(adjusted HR, 0.543; 95% CI, 0.080 3.705) vs. (1) vs. (adjusted HR, 2.146 95% CI, 0.499, 9.228)]. Overall, the interaction analysis revealed no interactive role in the association between baseline PP level and hospital mortality (P for interaction >0.05).

**Table 4:** The subgroup analysis for baseline pulse pressure on hospital mortality.

Subgroup	Total	Event (*n*, %)	Categories of the baseline pulse pressure, adjusted HR (95% CI)			P for interaction
			Low (≤56 mmHg)	Reference (57–68 mmHg)	High (≥69 mmHg)	
Sex						0.876
Male	389	73 (18.8)	1.923 (0.927, 3.99)	1	1.814 (0.899, 3.661)	
Female	304	69 (22.7)	1.629 (0.766, 3.467)	1	1.613 (0.769, 3.384)	
Myocardial infarction						0.907
No	635	121 (19.1)	1.836 (1.063, 3.168)	1	1.765 (1.042, 2.991)	
Yes	58	21 (36.2)	1.382 (0.148, 12.94)	1	1.144 (0.116, 11.28)	
Congestive heart failure						0.111
No	643	121 (18.8)	1.747 (1.024, 2.980)	1	1.459 (0.866, 2.461)	
Yes	50	21 (42.0)	3.767 (0.228, 62.26)	1	11.23 (0.826, 152.62)	
Hypertension						0.601
No	347	77 (22.2)	1.360(0.658, 2.812)	1	1.811 (0.890, 3.686)	
Yes	346	65 (18.8)	2.101 (0.978, 4.514)	1	1.142 (0.557, 2.338)	
Diabetes						0.154
No	605	117 (19.3)	1.912 (1.090, 3.353)	1	1.601 (0.920, 2.785)	
Yes	88	25 (28.4)	0.543 (0.080, 3.705)	1	2.146 (0.499, 9.228)	
Embolization of aneurysm						0.743
No	491	116 (23.6)	1.975 (1.093, 3.570)	1	1.940(1.087, 3.463)	
Yes	202	26 (12.9)	1.454 (0.421, 5.024)	1	1.333 (0.452, 3.929)	
GCS score						0.503
<8	219	81 (37)	1.084 (0.555, 2.117)	1	1.272 (0.668, 2.422)	
≥8	474	61 (12.9)	1.519 (0.575, 4.011)	1	1.952 (0.826, 4.614)	

The model was adjusted, if not stratified, for age, sex, ethnicity, RR, WBC, platelet, GCS score, Charlson Comorbidity Index, congestive heart failure, myocardial infarction, hypertension, sepsis, vasoactive drugs, nicardipine, nimodipine, embolization of aneurysms.

In the Supplementary analysis, we also examined the relationship between baseline PP levels, the 28 day mortality, and ICU mortality. Both 28 day and ICU mortality had a non-liner relationship with baseline PP levels (Supplemental Figure S1 Figure 2).

## Discussion

In this retrospective cohort study of ICU patients with non-traumatic SAH, we found a nonlinear association between the admission PP level and hospital mortality after adjusting for baseline covariates, with an inflection point at approximately 60 mmHg and minimal risk at 57 to 68 mmHg of the admission PP level.

The relationship between PP level in the acute phase of stroke and outcomes has been investigated in some studies. Grabska et al. and Su et al. examined PP levels and 30 day mortality, and they discovered that elevated PP levels were associated with an increased risk of 30 day mortality (11, 12). Domanski et al. reported that for every 10 mmHg increase in pulse pressure, there was an 11% increase in the risk of stroke and a 16% increase in the risk of all-cause mortality in elderly patients with systolic hypertension (5). A post-hoc analysis of 1,479 patients from the TAIST (Tinzaparin in Acute Ischaemic Stroke Trial) found that elevated PP upon admission among patients within 48 h of ischemic stroke was independently linked to mortality (odds ratio, 1.02; 95% CI, 1.01–1.03) (7). Vemmos et al. discovered that every 10 mm Hg increase in 24 h PP levels resulted in higher mortality (HR, 1.39; 95% CI, 1.04–1.86) for 1 year during the acute stroke period (9). According to our investigation, when the admission PP level was ≥105 mmHg, a 5 mm Hg increase in PP upon admission corresponded to a 39% increased risk of hospital mortality (HR, 1.39; 95% CI, 1.05–1.83) after non-traumatic SAH. In a study of 672 patients with non-traumatic cerebral hemorrhage (ICH), Jason et al. discovered that the higher PP levels (first 12 h mean PP) after admission were linked to increased in-hospital mortality (odds ratio, 3.0; 95% CI, 1.7–5.3) (8). However, there are few studies on the association between PP level and mortality in SAH patients. A previous study including 156 patients with SAH demonstrated no statistically significant association between admission PP and hospital mortality (13). Contrary to these results, our study demonstrated that a higher or lower PP upon admission was associated with an increased risk of hospital mortality. Similar results were seen among non-traumatic SAH patients with HF, hypertension, and embolization of an aneurysm, even if vasopressor requirements were considered. These results highlight the role of the pulsatile element of BP and the importance of ensuring constant perfusion and cardiac function on post-SAH outcomes. Also, it suggests that the importance of admission PP level extends beyond the non-surgical population alone. In support of our findings, a Taiwan prospective cohort study of 33,530 individuals aged >18 years found a U-shaped association between PP at admission and a 3 month unfavorable outcome in acute ischemic stroke (10). We also observed a threshold effect. The non-traumatic SAH patients with PP upon admission range of 57 to 68 mmHg were at the lowest risk of mortality. Our study suggests that maintaining PP goals lower than about 60 mmHg may not be adequate to preserve organ perfusion. However, compared to previous studies, first, our study assessing PP based on the non-traumatic SAH population was larger. Second, the disease severity in the study population may have been different as it was from the ICU. Third, the adjustment variables were also different, and we adjusted for several well-known outcome parameters, disease severity, ICU treatment, and comorbidities of common serious illnesses. Furthermore, this may have implications for SAH BP management and warrants further investigation.

The potential underlying mechanisms behind the close correlation between the admission PP level and hospital mortality in ICU patients with non-traumatic SAH are still unknown. Potential mechanisms are presented below. First, the PP level depends on the vascular elasticity of the systolic ejection dilation catheter arteries and the large arteries (27–31). Thus, the level of PP indirectly reflects the degree of arterial stiffness. Furthermore, increased PP is common in high-risk patients with large artery stiffness, aortic regurgitation, and increased systolic hypertension (6, 32). Second, elevated PP value might lead to greater cyclic strain that induces inflammatory endothelial cell responses, increasing the risk of inflammatory-related diseases such as atherosclerotic cardiovascular disease (33). Thirdly, recent studies have found that a high PP would produce non-steady shear stress forces. This non-steady shear might well result in oscillatory (i.e., reversing or ‘back-and-forth’) shear stress forces, particularly at arterial branch points during which blood flow is still non-steady due to branch point geometry (34). Fourth, a low PP may be indicative of lower CBF due to impaired cerebral autoregulation or reduced cerebral perfusion, which could increase the risk of cerebral vasospasm (35). Interestingly, we found an inconsistent relationship between PP and in-hospital mortality rates in SAH patients with and without diabetes. We conducted further investigation into this phenomenon. The increased risk of in-hospital mortality in diabetic patients with higher PP may be attributed to several factors. Firstly, diabetic patients often have multiple cardiovascular diseases and complications, such as hypertension, coronary heart disease, and heart failure, which may affect the relationship between PP and mortality. Secondly, diabetic patients often have pathological and physiological changes, such as endothelial dysfunction and vascular damage, which may lead to arterial stiffness and increased vascular resistance, thereby increasing the risk of cardiovascular events and death (29, 36). Additionally, metabolic abnormalities such as hyperglycemia and insulin resistance in diabetic patients may affect vascular contraction and relaxation, thereby influencing the magnitude of PP and its impact on cardiovascular events (37, 38). In conclusion, there are specific physiological and metabolic changes in the relationship between PP and in-hospital mortality in diabetic patients, which require further research to explore their specific mechanisms. Overall, the factors affecting PP mentioned above may increase the risk of adverse outcomes in SAH patients, and the exact mechanisms need to be investigated further.

Our study has several limitations. First, we utilized data from the ICU Medical Center in the United States, and because this was a single-center study, the results may not be completely representative of the general non-traumatic SAH patient population. Second, this was a retrospective cohort study with limitations such as the possibility of unmeasured confounders and missing data influencing the results. Furthermore, admission diagnoses are premised on ICD-9 and ICD-10 codes, which may undercount the number of patients suffering from non-traumatic SAH and related complications. As a result, we conducted a sensitivity analysis that included some interventions and common comorbidities. Fortunately, the sensitivity analysis result remained consistent. Third, because PP data were limited to baseline values at ICU admission, this study could only confirm the correlation between admission PP levels and hospital mortality without establishing a causal relationship. Four, we analyzed the first PP record collected during ICU admission, and therefore, the results are limited to a confined period during which PP was measured. In addition, the blood pressure values obtained from the MIMIC database may have been collected using equipment from different manufacturers. Therefore, there is no way to accurately standardize blood pressure measurement. However, each medical unit regularly calibrated monitors to prevent errors in medical practice.

## Conclusion

For patients with non-traumatic SAH, the association between baseline PP and risk of hospital mortality was non-linear, with an inflection point at 60 mmHg and a minimal risk at 57 to 68 mmHg (Q2) of baseline PP level. If further confirmed, our findings provides evidence for the early identified high risk of hospital mortality in non-traumatic subarachnoid hemorrhage population.

## Data availability statement

The original contributions presented in the study are included in the article/Supplementary material, further inquiries can be directed to the corresponding authors.

## Ethics statement

The studies involving human participants were examined and approved by Beth Israel Deaconess Medical Center. To protect patient privacy, all data were de-identified; therefore, the Ethical Committee of the Beth Israel Deaconess Medical Center waived the requirement for informed consent. Written informed consent for participation was not required for this study in accordance with the national legislation and the institutional requirements.

## Author contributions

HZ and XW conceived the study idea. JL and HZ analyzed the data, reviewed the literature, and wrote the first draft. LJ and SW critically reviewed and edited the manuscript and approved the final version. All authors contributed to the article and approved the submitted version.

## Conflict of interest

The authors declare that the research was conducted in the absence of any commercial or financial relationships that could be construed as a potential conflict of interest.

## Publisher’s note

All claims expressed in this article are solely those of the authors and do not necessarily represent those of their affiliated organizations, or those of the publisher, the editors and the reviewers. Any product that may be evaluated in this article, or claim that may be made by its manufacturer, is not guaranteed or endorsed by the publisher.

## Footnotes

^1^http://www.r-project.org
