# Supplementary figures and images for: Association between baseline pulse pressure and hospital mortality in non-traumatic subarachnoid hemorrhage patients: a retrospective cohort study
Source: Front Neurol. 2023 Jul 17;14:1176546. doi: 10.3389/fneur.2023.1176546 (PMC10389704; doi:10.3389/fneur.2023.1176546)

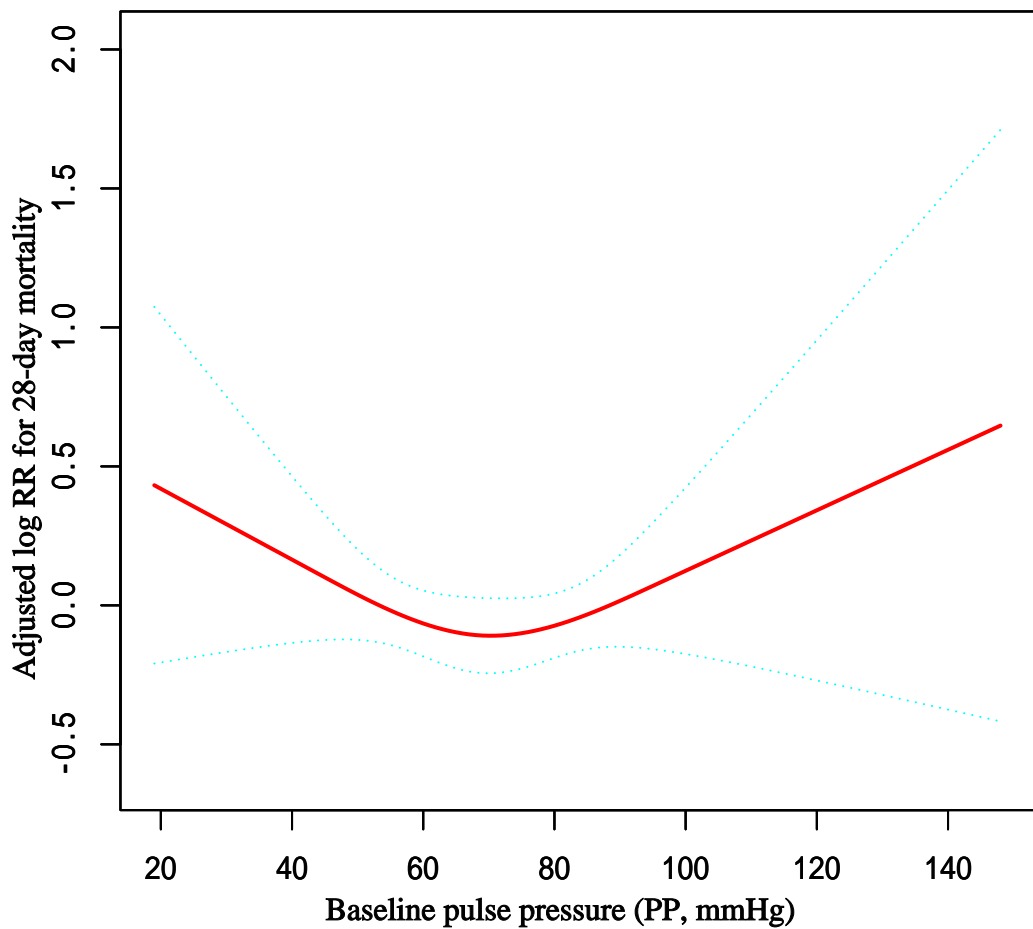

Supplement: Supplementary file 1 [file Data_Sheet_1.PDF]

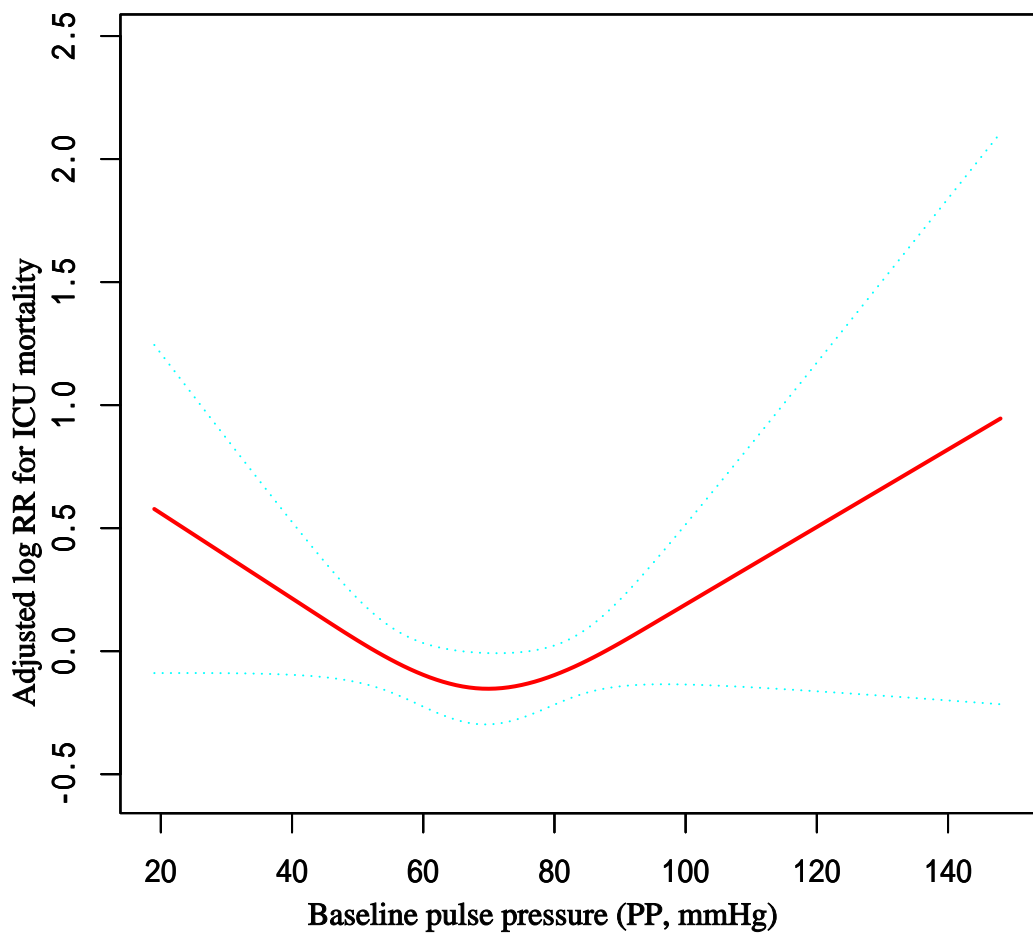

Supplement: Supplementary file 2 [file Data_Sheet_2.PDF]
