# Supplementary material for: Association between baseline pulse pressure and hospital mortality in non-traumatic subarachnoid hemorrhage patients: a retrospective cohort study
Source: Front Neurol. 2023 Jul 17;14:1176546. doi: 10.3389/fneur.2023.1176546 (PMC10389704; doi:10.3389/fneur.2023.1176546)
Supplement: Supplementary file 3 [file Table_1.DOC]

**Supplemental TABLE**

| **Supplemental TABLE 1 |** Details of missing values | | |
| --- | --- | --- |
| **Variables** | **The number of missing values** | **The percent of missing values** |
| RR | 2 | 0.1% |
| BUN | 1 | 0.1% |
| creatinine | 1 | 0.1% |
| platelet | 2 | 0.1% |
| WBC | 1 | 0.1% |

Note: RR, respiratory rate; WBC, white blood cell; BUN, blood urea nitrogen;

Supplemental TABLE 2| Univariate Cox regression analyses for hospital mortality in patients with non-traumatic SAH.

| **Variable** | **HR 95 CI%** | **P value** |
| --- | --- | --- |
| **Age, years** | 1.03 (1.02,1.04) | < 0.001 |
| **Female,** n(%) | 1.07 (0.77,1.5) | 0.679 |
| **White race,** n(%) | 1.92 (1.38,2.69) | < 0.001 |
| **Vital signs** |  |  |
| SBP, mmHg | 0.99 (0.99,1.01) | 0.395 |
| DBP, mmHg | 0.99 (0.98,1.01) | 0.248 |
| MBP, mmHg | 0.98 (0.96,1.00) | 0.058 |
| Heart rate, beats/minute | 1.02 (1.00,1.03) | 0.008 |
| RR, times/minute | 1.11 (1.07,1.16) | < 0.001 |
| Temperature, °C | 0.79 (0.59,1.06) | 0.117 |
| SpO2, % | 0.96 (0.87,1.05) | 0.336 |
| **Comorbidities, n (%)** |  |  |
| Myocardial infarction | 1.78 (1.12,2.83) | 0.015 |
| Congestive heart failure | 1.89 (1.19,3.02) | 0.007 |
| Chronic pulmonary disease | 1.29 (0.86,1.93) | 0.225 |
| Diabetes | 1.3 (0.84,2.00) | 0.242 |
| Hypertension | 0.80 (0.57,1.12) | 0.188 |
| paraplegia | 0.99 (0.64,1.54) | 0.964 |
| Sepsis | 1.57 (1.06,2.34) | 0.026 |
| Charlson comorbidity index | 1.17 (1.10,1.24) | < 0.001 |
| **Laboratory results** |  |  |
| WBC,10^9^/L | 1.03 (1.02,1.05) | < 0.001 |
| Platelets,10^9^/L | 0.99 (0.99,0.10) | 0.009 |
| Hemoglobin, g/dl | 0.92 (0.85,1.00) | 0.059 |
| Glucose, mg/dl | 1.01 (1.01,1.01) | < 0.001 |
| Sodium, mg/dl | 1.08 (1.06,1.11) | < 0.001 |
| Potassium, mg/dl | 1.30(1.12,1.50) | < 0.001 |
| Bun, mg/dL | 1.03 (1.03,1.04) | < 0.001 |
| Creatinine, mg/dL | 1.18 (1.11,1.26) | < 0.001 |
| **Therapy, n (%)** |  |  |
| Norepinephrine | 1.99 (1.42,2.79) | < 0.001 |
| Vasopressin | 2.52 (1.72,3.70) | < 0.001 |
| Dopamine | 2.40 (1.06,5.45) | 0.036 |
| Nicadipine | 0.68 (0.48,0.96) | 0.028 |
| Nimodipine | 0.47 (0.34,0.66) | < 0.001 |
| Embolization of aneurysm | 0.52 (0.34,0.80) | 0.003 |
| Clipping of aneurysm | 0.61 (0.25,1.50) | 0.283 |
| **Scoring systems** |  |  |
| GCS | 0.92 (0.88,0.96) | < 0.001 |

Abbreviations: SBP, systolic blood pressure; DBP, diastolic blood pressure; MBP, mean blood pressure; RR, respiratory rate; SpO2, percutaneous oxygen saturation; WBC, white blood cell; GCS, Glasgow coma score; ICU, Intensive care unit; SAH, subarachnoid hemorrhage
